# Highly Efficient Methods with a Generalized Linear Mixed Model for the Quantitative Trait Locus Mapping of Resistance to Columnaris Disease in Rainbow Trout (*Oncorhynchus mykiss*)

**DOI:** 10.3390/ijms252312758

**Published:** 2024-11-27

**Authors:** Yuxin Song, Zhongyu Chang, Ao Chen, Yunfeng Zhao, Yanliang Jiang, Li Jiang

**Affiliations:** 1 Wuxi Fisheries College, Nanjing Agricultural University, Wuxi 214081, China; 2 Research Centre for Aquatic Biotechnology, Chinese Academy of Fishery Sciences, Beijing 100141, China; czy821ouc@163.com (Z.C.); m19503860141@163.com (A.C.); zhaoyf@cafs.ac.cn (Y.Z.);; 3 Beijing Key Laboratory of Fishery Biotechnology, Chinese Academy of Fishery Sciences, Beijing 100141, China; 4 Key Laboratory of Aquatic Genomics, Ministry of Agriculture and Rural Affairs, Beijing 100141, China; 5 College of Fisheries and Life Science, Shanghai Ocean University, Shanghai 201308, China

**Keywords:** linear mixed models, Columnaris disease, rainbow trout, genome-wide association studies

## Abstract

Linear mixed models (LMMs) are commonly used in genome-wide association studies (GWASs) to evaluate population structures and relatedness. However, LMMs have been shown to be ineffective in controlling false positive errors for the analysis of resistance to Columnaris disease in Rainbow Trout. To solve this problem, we conducted a series of studies using generalized linear mixed-model association software such as GMMAT (v1.4.0) (generalized linear mixed-model association tests), SAIGE (v1.4.0) (Scalable and Accurate Implementation of Generalized mixed model), and Optim-GRAMMAR for scanning a total of 25,853 SNPs. Seven different SNPs (single-nucleotide polymorphisms) associated with the trait of resistance to Columnaris were detected by Optim-GRAMMAR, four SNPs were detected by GMMAT, and three SNPs were detected by SAIGE, and all of these SNPs can explain 8.87% of the genetic variance of the trait of resistance to Columnaris disease. The heritability of the trait of resistance to Columnaris re-evaluated by GMMAT was calibrated and was found to amount to a total of 0.71 other than 0.35, which was seriously underestimated in previous research. The identification of *LOC110520307*, *LOC110520314*, and *LOC110520317* associated with the resistance to Columnaris disease will provide us more genes to improve the genetic breeding by molecular markers. Finally, we continued the haplotype and gene-based analysis and successfully identified some haplotypes and a gene (*TTf-2*) associated with resistance to Columnaris disease.

## 1. Introduction

Genome-wide association analysis (GWAS) is a statistical method utilized for the detection of quantitative trait loci (QTLs) that are associated with complex traits. It involves the identification of single-nucleotide polymorphisms (SNPs) throughout the entire genome, and the subsequent association analysis between these SNPs and the phenotype of interest. Over the past decade, GWAS has been successfully applied to identifying genetic markers associated with major quantitative traits in both plants and animals, especially those of economic importance [1,2]. Compared to terrestrial livestock, major QTLs in aquaculture may be more prominent, possibly due to the diversity of life history strategies and early stages of domestication of aquatic species [3,4]. In recent years, significant progress has been made in genome-wide association analysis in aquaculture economic animals, particularly in the study of traits of disease resistance [4,5,6,7,8]. Genome-wide association studies (GWASs) were utilized to identify the gene loci associated with resistance to putrefactive body disease in large yellow croaker [6]. A single-nucleotide polymorphism (SNP) locus that confers resistance to Streptococcus agalactiae was discovered in oval-shaped marine fish [9]. Six gene loci associated with resistance to Nervous Necrosis Virus (NNV) infection in Asian sea bass were identified [10]. The quantitative trait loci (QTLs) related to salmon resistance in Rainbow Trout were determined [11]. The candidate genes involved in viral replication and immune response in Rainbow Trout were confirmed [12]. The QTL for resistance to Vibrio anguillarum in Rainbow trout was verified [13].

A complex population structure leading to population stratification is an important confounding factor in GWAS [14,15,16]. With the development of computationally efficient algorithms, linear mixed models (LMMs) have become a popular approach to controlling population stratification and familial or unknown relatedness in GWASs [17,18]. However, GWAS was utilized to investigate some binary traits in aquatic animals, such as the trait of disease resistance in fish and the trait of sex, in which LMMs typically assume constant residual variance, which is not realistic in the presence of covariates [19]. Therefore, fitting binary traits using LMMs in the presence of a complex population structure may result in failure to control the type I error rate and produce incorrect *p*-value estimates [20]. To account for the random polygenic effects, generalized linear mixed models (GLMMs) are needed to improve the power of QTNs detection for binary traits [21]. The exact steps for solving the GLMM are similar to those for solving the LMM: the first step is to calculate the genomic relationship matrix, the second step is to estimate the variance components of the generalized linear mixed model, and the third step is to perform association tests for each marker.

Given that the second step of the variance component estimation in GLMM is computationally more complex than LMM, and the third step for logit regression in high-throughput data is also computationally more complex than linear regression, simplified GLMM algorithms are needed for genome-wide association analysis [20]. To simplify GLMM calculations, researchers have proposed the following algorithms: the generalized linear mixed model association test (GMMAT) method based on the GLMM extension of EMMAX [20,22]; Scalable and Accurate Implementation of Generalized mixed model (SAIGE), which draws on BOLT-LMM [23] and GRAMMAR-Gamma [24] methods and extends them to generalized linear mixed models [25]; and the Optim-GRAMMAR method, which extends the GRAMMAR method to binary traits [26] and optimizes the genetic power of the genome to control false negatives. Subsequently, building upon the foundation of GMMAT, the variant-set mixed model association tests (SMMAT) was designed for both continuous and binary traits within the generalized linear mixed-model framework. These tests are tailored for the analysis of variant sets such as haplotypes and genes [27].

Columnaris is a disease symptom that commonly affects fish and is caused by the aerobic, rod-shaped, Gram-negative bacterium Flavobacterium columnare. Consequently, there is great interest in selections for improved resistance to this disease in aquaculture breeding programs. In previous studies, GCTA software (v1.94.1 Linux) was used to perform genetic mapping and estimate genetic parameters for resistance to Columnaris disease among two cultured Rainbow Trout (*Oncorhynchus mykiss*) populations using the MLM-LOCO model [4]. The estimated heritability of this trait is approximately 0.35 in both populations, and major and minor quantitative trait loci were identified on chromosome 2 and chromosome 3, respectively, with a shared quantitative trait locus on Omy05 explaining a total of 3% of the genetic variation in both populations.

We compared the performances of the GMMAT, SAIGE, and Optim-GRAMMAR methods with GLMM in terms of QTN mapping accuracy and detection power in both populations. At the same time, the advantages and disadvantages of these methods in genome-wide analysis of the trait of resistance to Columnaris disease were summarized.

## 2. Results

### 2.1. Genome-Wide Association Analysis

**Figure 1:**
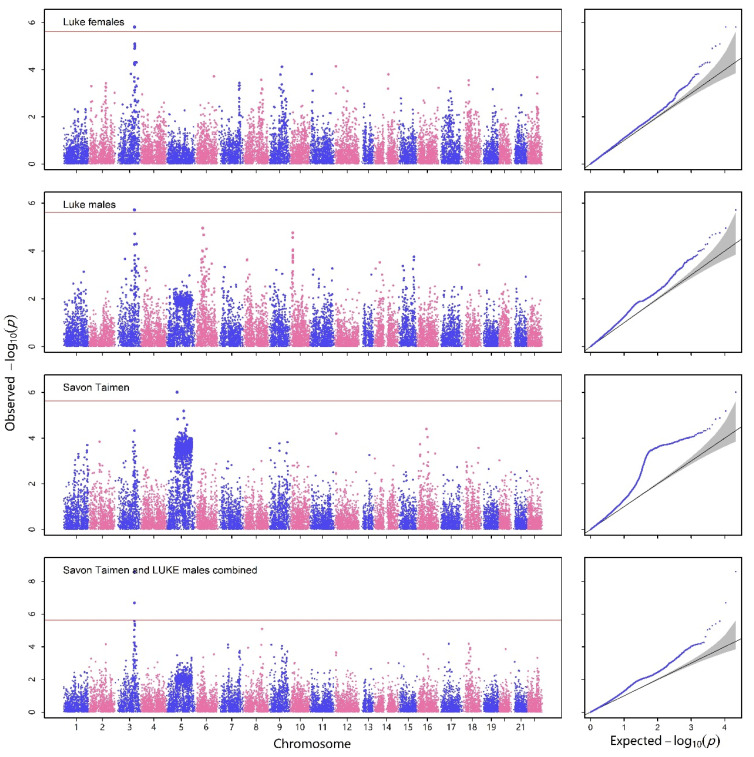
Manhattan (**left**) and Q-Q plots (**right**) of genome-wide association analysis using the method of MLMA-LOCO for different population subsets, including LUKE female fish samples, LUKE male fish sample, and Savon Taimen female fish sample; all fish samples were combined, and Savon Taimen and LUKE males were combined. The horizontal reference lines in Manhattan plots represent Bonferroni correction thresholds at a significance level of 5%. The every blue and pink dots represents a SNP.

**Figure 2:**
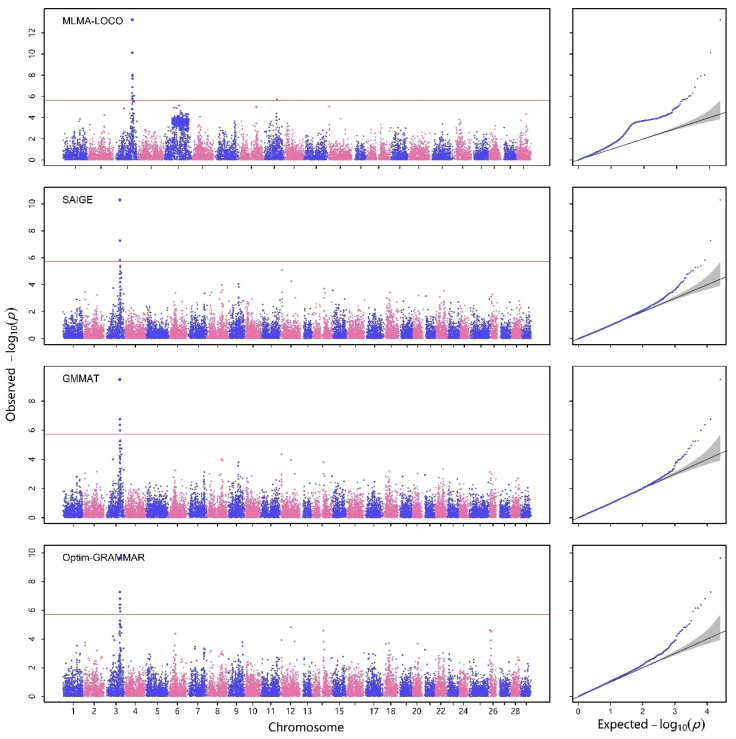
Manhattan (**left**) and Q-Q plots (**right**) obtained by MLMA-LOCO, SAIGE, GMMAT, and Optim-GRAMMAR with all of the samples. The horizontal reference lines in Manhattan plots represent Bonferroni correction thresholds at a significance level of 5%. The every blue and pink dots represents a SNP.

First, we replicated the results using the MLMA-LOCO analysis method as reported in the original study in Figure 1 and Figure 2. The majority of results in the Manhattan plots are consistent with the original article, except for the Savon Taimen and LUKE males combined population subset, where the locus on chromosome 5 was not detected. However, the Q-Q plot showed deviations from the expected line, indicating a risk of false positives in this population. Subsequently, we tested three methods based on GLMM, namely SAIGE, GMMAT, and Optim-GRAMMAR, as shown in Figure 2. The Q-Q plots for these methods were all consistent with the expected line, indicating good statistical properties. The most maximum numbers of QTNs by Optim-GRAMMAR were detected, amounting to seven QTNs, followed by GMMAT with four QTNs detected. The minimum quantity of QTNs was detected by SAIGE, with only three identified.

We list the seven most significant SNPs associated with the trait of mortality in Table 1. All of these markers were located on chromosome 3, and we calculated the variance explained by each SNP using the original formula. We found that there were significant differences in the results obtained by Optim-GRAMMAR and SAIGE compared to those obtained by GMMAT. Specifically, GMMAT explained the highest total variance, followed by SAIGE, while Optim-GRAMMAR explained the least total variance.

In our analysis of real genetic data, three genetic components of SNPs, haplotype alleles, and genes, as illustrated in Figure 2, were investigated. It is important to note that SNPs are a subset that falls within the broader category of haplotype alleles and genes. The partitioning of haplotypes was carried out using HaploView 4.2 software, and gene information was sourced from the NCBI database. Ultimately, the location of the haplotype was found on chromosome 3, which contained the associated SNP with the trait. Additionally, the genes of *TTN*, *CWC22*, *neurod1*, and *rubcnl* were found to be associated with Columnaris disease.

### 2.2. Heritability

We observed a super-high level of genetic heritability in two populations. The detailed data is in Table 2. The GLMM model estimates for LUKE showed *h*^2^ = 0.71, SD = 0.04, and for Savon Taimen, *h*^2^ = 0.65, SD = 0.02. The overall estimate for genetic heritability across both populations was *h*^2^ = 0.71, SD = 0.01.

## 3. Discussion

Rainbow Trout is the main cold-water economic variety, and the harm caused by the disease to the industry cannot be ignored. Red mark syndrome (RMS), which is also called cold water strawberry disease [28], is only witnessed in fish within an environment where the water temperature is below 15 °C. It is a skin-related condition associated with Rickettsia-like organism (RLO) infection, and it emerges in Rainbow Trout under particular circumstances [29]. Commonly referred to as strawberry disease, RMS is recognized as a non-fatal condition that typically appears during the winter and spring seasons [30]. Apparently, this disease has been named warm water strawberry disease and rash [31]. RMS initially emerged in a single Rainbow Trout farm in Scotland and then simultaneously spread throughout the rest of the United Kingdom [32]. Additionally, the disease spread sporadically to numerous countries, including those in continental Europe, the USA, Chile, and Japan, thereby causing multiple problems [33]. The affected fish are all individuals with a weight of more than 500 g that meet the market specification. There are seldom individuals under this specification suffering from this disease [34]. This has resulted in substantial economic losses. Meanwhile, the fish individuals with this disease do not exhibit anorexia or growth inhibition [35]. Moreover, despite having a relatively high morbidity rate of around 5% to 50%, this disease is not associated with fish mortality [36].

Flavobacterium columnare (*F. columnare*), the causative agent of Columnaris disease, belongs to the family of Flavobacteriaceae [37,38,39]. *F. columnare* is distributed worldwide in fresh water sources and may infect many different wild and cultured freshwater fish species, such as (but not limited to) carpchannel catfish, goldfish, eel, perch, salmonids, and tilapia [37,40,41,42,43,44,45]. Many tropical freshwater aquarium fishes are also infected with this bacterium [40,42]. In the channel catfish (*Ictalurus punctatus*) industry in the United States, F.columnare is the second most prevalent bacterium, after Edwardsiella ictaluri, to cause disease and mortality [45,46], with yearly losses estimated at USD 30 million [47]. This organism can also be a part of the bacteria microbiota of freshwater fish, eggs, and the rearing waters the fish live in [48].

The binary trait of resistance to Columnaris disease in Rainbow Trout was genetically analyzed by the GLMM method, and new QTNs with better fitness were found. Compared with the previous study of this trait by the MLM-LOCO method, we found that the GLMM mixed model can effectively control false positive errors in GWAS for binary traits. On the other hand, even when considering fixed effects and using LMM to control the polygenic effects of binary traits, false positive errors may still occur in the presence of population stratification, especially in complex animal and plant breeding populations with complicated pedigree structures, which may face a higher risk of such errors.

*TTN*, *CWC22*, and *RUBCN1* genes have important biological roles and are linked to the development of certain diseases [49,50,51,52,53]. In the NCBI database, rubcnl assumes the function of protein coding in fishes like Danio rerio and Larimichthys crocea. Although they are not directly associated with resistance to Columnaris disease in Rainbow Trout, mutations in these genes may affect the immune system of animals, gut health, growth, and metabolism, making them more susceptible to infection. Studying these genes can help improve the aquaculture and understanding of the molecular characters of disease resistance in Rainbow Trout. Additionally, the identification of *LOC110520307*, *LOC110520314*, and *LOC110520317* associated with the resistance to Columnaris disease will provide us with more genes to improve the genetic breeding by molecular markers. Based on the gene localization results, a significant association was identified between *TTf-2* and the target traits. Mutation in the gene encoding human *TTF-2* is associated with thyroid agenesis, cleft palate, and choanal atresia.

As shown in Figure 1, the rates of false positive were gradually controlled as the population subsets were stratified. However, the incomplete resolution of issues when using a group as covariates, especially in the presence of genetic differentiation between different populations, may have contributed to the false positive performance of MLMA-LOCO [20]. Furthermore, as shown in Figure 1, we observed a slight increase in false positives, even in single-sex subpopulations. The application of LMM may lead to erroneous Type I error rates when heterogeneity in disease risk or differential case-control ratios exist in the data, and the homoscedasticity assumption of LMM may be ignored when interpreting binary traits, leading to misleading results. Therefore, caution is needed when using an LMM-based method, such as MLMA-LOCO, and alternative methods, such as GLMM-based methods, should be considered for genetic association studies in complex populations. We compared three mixed-model association methods based on GLMMs: GMMAT, SAIGE, and Optim-GRAMMAR, in terms of their performance in the estimation of the SNP effect, computational efficiency, and power to detect QTNs. All three methods exhibited excellent statistical properties, without any false positives or negatives. GMMAT considered the binary nature of the dependent variable. It first estimated variance components using a GLMM and then built a score statistic for each SNP, controlling false positives in the presence of population stratification. However, GMMAT had a heavier computational burden for computing SNP effects [20]. Nonetheless, these effects could be used as a reference, and Optim-GRAMMAR provided relatively accurate effects compared to SAIGE. SAIGE employed the same variance component estimation algorithm and association statistics as GMMAT but utilized a subset of markers sampled randomly from the genome to calculate the genetic relationship matrix (GRM), thereby simplifying the computation process. Additionally, SAIGE incorporates a modified score statistic to account for unbalanced case–control ratios through the use of the SPA correction test [25]. Although SAIGE exhibited good statistical properties in human dataset such as UK Biobank, recent studies have suggested that it may produce some false positives when samples are in highly correlated or complex structures [26]. Optim-GRAMMAR demonstrated the highest computational efficiency and power to detect QTNs, as described in its original paper [4]. Optim-GRAMMAR enables fast whole-genome association analysis and effectively avoids the problem of tightened association statistics and inaccurate estimation of genetic parameters in complex diseases. Compared to other GLMM-based whole-genome association analysis methods, Optim-GRAMMAR requires only a few iterations of solving equations based on genome-wide best linear unbiased prediction (GBLUP) using sampled markers and simple logistic regression for association testing, greatly enhancing the computational efficiency of GLMM association analysis. Among existing GLMM-based association methods, Optim-GRAMMAR has the lowest computational complexity for binary trait by mixed-model association testing.

Optim-GRAMMAR represents a significant breakthrough in the analysis of genomic data, with its capabilities of joint analysis and improved statistical power in more accurate and reliable QTN detection. Its implementation in R language further enhances its accessibility to both researchers and practitioners. We highly recommend Optim-GRAMMAR for QTNs’ mapping, especially in complex traits in breeding populations of plants and animals. However, for those interested in SNP effect values and genetic power, we recommend GMMAT for genetic analysis or a combination of the two methods. Specifically, we suggest using GMMAT for genetic power analysis, Optim-GRAMMAR for gene mapping, and then GMMAT for calculating effect values for significant SNPs. By combining these methods, researchers can achieve more accurate results by comprehensively utilizing the methods of whole-genome wide association analysis.

## 4. Materials and Methods

### 4.1. Animal Populations, Family Structure, Collection of Phenotype

The samples for this study were collected from two populations of Rainbow Trout. The first population, Savon Taimen, consisted of 1450 offsprings and 104 parents from the Savon Taimen fish production company. The second population, LUKE, consisted of 3055 offsprings and 81 parents. The two groups, although their ancestors were not from the same farm, had offsprings that were raised on the same farm [4].

DNA was extracted from these samples and genotyped using the commercial 57K SNP Axiom Trout genotyping array, and we exported the resulting genotypes as PLINK format .ped and .map files for further analysis. After quality control, 25,853 high-quality SNPs classified as being polymorphic at a high resolution were included in the analysis. It should be noted that the data utilized in this study were downloaded from https://doi.org/10.6084/m9.figshare.19323506 [4].

The phenotype of interest in this study was the trait of resistance to Columnaris disease, which was represented by a binary trait indicating whether the offsprings had died or were dying due to the disease. Sex and pool location were considered as the fixed effects in the analysis. All samples from the Savon Taimen population were females, so sex determination was not necessary. For the LUKE population, seven SNPs related to sex were used as molecular markers to determine the sex of the offspring. The sensitivity and specificity of these markers were evaluated using the known sexes of the LUKE parents. There were 1341 males and 1337 females among the LUKE population after molecular identification. The populations were located in different pools, with Savon Taimen in pools 48, 49, and 50, and LUKE in pools 51, 52, and 53. Therefore, the pool location was considered as another fixed effect in the analysis.

### 4.2. Model of Genome-Wide Association Analyses

In addition to continuous traits, genome-wide association studies (GWASs) are also commonly used to analyze dichotomous traits such as the trait of disease and sex. Complex diseases are typically considered to be quantitative traits controlled by multiple genes with small effects. Therefore, when mapping quantitative trait nucleotides (QTNs) for dichotomous phenotypes, the traditional approach is to use the logistic regression in generalized linear models (GLMs) rather than simple linear regression models. Although logistic regression can correct fixed effects with covariates, inflation of test statistics still occurs in the GWAS analysis of dichotomous traits of disease with complex structured populations. In addition, the assumption of constant residual variance in linear mixed models (LMMs) contradicts the distribution of dichotomous traits, and, sometimes, an LMM is unable to control false positive errors caused by population stratification in GWASs for dichotomous traits. Moreover, the parameters estimated by linear models cannot be used for statistical inference of dichotomous traits. Therefore, to consider random polygenic effects, the following generalized linear mixed model (GLMM) is introduced to improve the mapping power of QTNs for traits of disease and sex.

$$\mathrm{logit}\left(\pi \right)=X\beta +Za+g$$

This equation represents the probability of the dichotomous phenotype for the subject, where Xβ represents the fixed effects regression term, which includes the effects of tank and sex in this study, Za represents the regression term for the marker genotype effect to be tested, and g represents the residual polygenic effects.

### 4.3. Estimation of Heritability

To test the null hypothesis H0: a = 0, it is necessary to fit a logistic mixed-effects model with a null hypothesis.

$$\mathrm{logit}\left(\pi_{0}\right)=X\beta +g$$

To fit the formula using the penalized quasi-likelihood (PQL) method, we specifically define $W=\pi_{0}(1-\pi_{0})$ and $y^{*}=X\beta +g+W^{-1}(y-\pi )$ to represent the liability of binary traits. Under the null hypothesis H0: a = 0, we use the computationally efficient AI-REML algorithm to iteratively fit a generalized linear mixed-effects model, which was implemented in GMMAT software. The estimates for fixed effects and random effects can be updated along with a working vector until convergence is achieved for the heritability of the trait.

### 4.4. Statistical Methods

#### 4.4.1. MLMA-LOCO

We applied the MLMA-LOCO method by GCTA software [54] and the same model as described in the original article. Following the approach in the original article, we performed GWASs on different population subsets, including LUKE female samples, LUKE male samples, and Savon Taimen female samples; all fish samples were combined in this study.

#### 4.4.2. GMMAT

After the estimation of heritability and the association testing for each SNP to be performed, the weights of all SNPs were fixed to the weights fitted by the null model. This method is similar to the EMMAX method in LMMs, where only one GLMM model is fitted for each GWAS, greatly reducing the computational cost of GWASs for binary traits with exact generalized linear-mixed models. The GMMAT method was implemented by the R/GMMAT package.

#### 4.4.3. SAIGE

To execute the SAIGE method, the two main steps are as follows: Step 1, to estimate variance components and other model parameters by fitting an invalid generalized linear mixed model; and Step 2, to use the SPA to correct the association test statistics. Step 1 uses the AI-REML algorithm with the same computational efficiency as GMMAT to iteratively estimate the model parameters. Compared with GMMAT, SAIGE directly uses the original genotypes with no need for pre-computing the GRM. Furthermore, to further reduce memory usage during model fitting, the original genotype data are stored in a binary vector and calculated directly when needed, without the need to store the GRM matrix. Finally, SAIGE corrects the test statistics using the SPA method to obtain *p*-values that are more accurate than those based on the normal distribution. The SAIGE method was implemented by the R/SAIGE package.

#### 4.4.4. Optim-GRAMMAR

Optim-GRAMMAR involves incorporating genomic breeding values (GBVs) estimated in advance as a known predictor in genomic logit regression. This is achieved by regulating downward genomic heritability to control false negative errors that may arise, avoiding direct estimation of variance components in complex GLMMs. Specifically, the optimization steps are as follows:

1. Set an initial genomic heritability in the open interval of 0 to 1.
2. Solve for the genomic breeding values g using the BLUP equation.
3. Infer the genetic effects of each SNP statistically.
4. Calculate the mean of the genome-wide chi-square statistics for each SNP.
5. Plot the Q-Q plot of the genome-wide statistic probabilities.
6. Update the genomic heritability using Brent’s method.
7. Repeat steps 2–6 until the genomic control value approaches 1 or a satisfactory Q-Q plot is obtained.

To enhance the statistical power of QTN detection, the Optim-GRAMMAR method carries out a joint analysis of candidate QTNs. The implementation of this method was performed using the R language (v4.0.0).

#### 4.4.5. Variant Set GWAS Analysis

**Figure 3:**
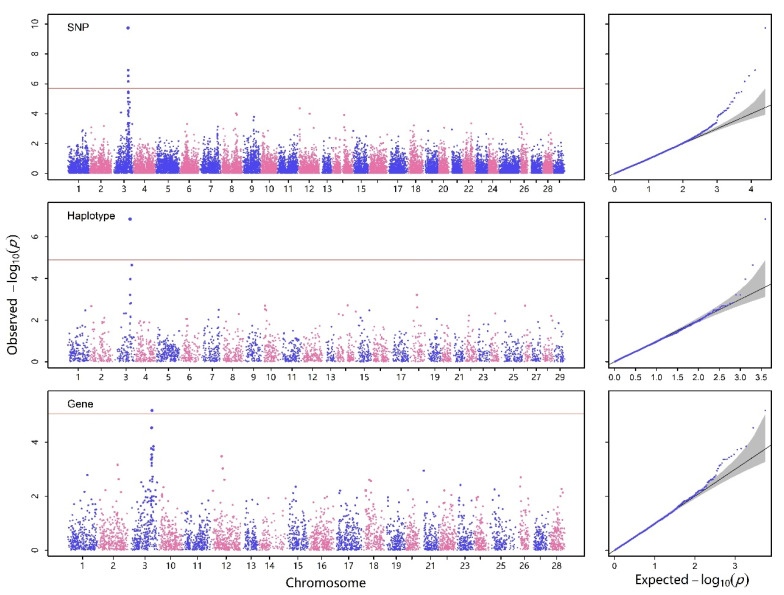
Manhattan (**left**) and Q-Q plots (**right**) of three genetic units with all of the samples. The horizontal reference lines in Manhattan plots represent Bonferroni correction thresholds at a significance level of 5%. The every blue and pink dots represents a SNP.

Finally, we employed variant set GWAS analysis to enhance the detection power of QTNs. The results of the analysis are available at Figure 3. Initially, we conducted a SMMAT analysis on this binary trait. Subsequently, we performed multivariate regression analysis, constraining the number of each variant set to within the total sample size. We employed a specific equation to from the same GLMM:

$$\mathrm{logit}\left(\pi \right)=X_{\mathrm{variant}\;\mathrm{set}}\beta_{\mathrm{variant}\;\mathrm{set}}+Za+g$$

In the multivariate regression equation, the regression terms for the markers being tested are evaluated. Due to the large sample size, we ultimately utilized the SMMAT-S test statistic mentioned in the original text.

## 5. Conclusions

Flavobacterium columnare is a well-known pathogen that usually leads to infectious disease in freshwater fish farming, where the disease produces an acute response. Eventually, scalable and acute mortality often arises in a short period of time. So, the dissection for a genetic basis of decreased mortality is valuable. We conducted a series of studies using generalized linear mixed-model association such as GMMAT, SAIGE, and Optim-GRAMMAR for association analysis between the SNP variants and the phenotype that has a higher statistical power and achieved the control of false positive errors. More associated SNPs and genes were found to be the future marker for resistance to bacterium infection of F.columnare in the breeding process, and the heritability was re-evaluated by GMMAT. All of these advances will speed up our genetic breeding of Rainbow Trout.

## Figures and Tables

**Table 1:** The most significant SNPs detected for association with the trait of resistance to Columnaris disease by GMMAT, Optim-GRAMMAR, and SAIGE.

	Chr	Pos	SNP	Gene	Freq	GMMAT BETA	Variance Explained	Optim-GRAMMAR BETA	Variance Explained	SAIGE BETA	Variance Explained	*p*-Value
**1**	3	64017036	AX-89974385	*LOC110520307 & TTN*	0.3319	−0.4542	1.8990	0.3189	0.9359	0.4859	2.1728	2.34 × 10^−10^
**2**	3	64032477	AX-89953544	*LOC110520307 & TTN*	0.2798	−0.4009	1.3444	0.2850	0.6793	0.4342	1.5768	5.29 × 10^−8^
**3**	3	64390419	AX-89933339	*LOC110520314 & cwc22*	0.4608	−0.3649	1.3736	0.2385	0.5866	0.3284	1.1121	4.09 × 10^−7^
**4**	3	64778731	AX-89959970	*LOC110520317*	0.3100	0.3834	1.3050	−0.2717	0.6554	−0.3374	1.0107	1.55 × 10^−7^
**5**	3	64779737	AX-89973040	*LOC110520317 & neurod1*	0.1792	−0.3836	0.8989	0.2911	0.5175	0.3882	0.9203	7.06 × 10^−7^
**6**	3	64781151	AX-89919724	*LOC110520317*	0.2702	0.3569	1.0427	−0.2654	0.5766	−0.3377	0.9335	7.07 × 10^−7^
**7**	3	65733733	AX-89976032	*rubcnl*	0.2917	−0.3440	1.0148	0.2468	0.5223	0.3417	1.0013	1.19 × 10^−6^

Chr is the number of the chromosome; Pos is the site of the SNP on a physical map of the genome; Freq represents the frequency of the SNP allele at the given position within the studied population.

**Table 2:** Heritability (*h*^2^) estimates of mortality in Savon Taimen, LUKE, and both populations using the GLMM model.

Population	*h* ^2^	SD
**Savon Taimen**	0.65	0.02
**LUKE**	0.71	0.04
**Total**	0.71	0.01

*h*^2^ is the heritability of the trait; SD is the standard difference.

## Data Availability

Data can be downloaded from Figshare at the following address: https://doi.org/10.6084/m9.figshare.19323506.
